# Quantitative characterization and analysis of the dynamic NF-κB response in microglia

**DOI:** 10.1186/1471-2105-12-276

**Published:** 2011-07-05

**Authors:** Patrick W Sheppard, Xiaoyun Sun, John F Emery, Rona G Giffard, Mustafa Khammash

**Affiliations:** 1 Department of Mechanical Engineering, University of California, Santa Barbara, Engineering II Bldg., Santa Barbara, CA, 93106-5070, USA; 2 Department of Anesthesia, Stanford University School of Medicine, Stanford, CA, 94305-5117, USA

## Abstract

**Background:**

Activation of the NF-κB transcription factor and its associated gene expression in microglia is a key component in the response to brain injury. Its activation is dynamic and is part of a network of biochemical species with multiple feedback regulatory mechanisms. Mathematical modeling, which has been instrumental for understanding the NF-κB response in other cell types, offers a valuable tool to investigate the regulation of NF-κB activation in microglia at a systems level.

**Results:**

We quantify the dynamic response of NF-κB activation and activation of the upstream kinase IKK using ELISA measurements of a microglial cell line following treatment with the pro-inflammatory cytokine TNFα. A new mathematical model is developed based on these data sets using a modular procedure that exploits the feedback structure of the network. We show that the new model requires previously unmodeled dynamics involved in the stimulus-induced degradation of the inhibitor IκBα in order to properly describe microglial NF-κB activation in a statistically consistent manner. This suggests a more prominent role for the ubiquitin-proteasome system in regulating the activation of NF-κB to inflammatory stimuli. We also find that the introduction of nonlinearities in the kinetics of IKK activation and inactivation is essential for proper characterization of transient IKK activity and corresponds to known biological mechanisms. Numerical analyses of the model highlight key regulators of the microglial NF-κB response, as well as those governing IKK activation. Results illustrate the dynamic regulatory mechanisms and the robust yet fragile nature of the negative feedback regulated network.

**Conclusions:**

We have developed a new mathematical model that incorporates previously unmodeled dynamics to characterize the dynamic response of the NF-κB signaling network in microglia. This model is the first of its kind for microglia and provides a tool for the quantitative, systems level study the dynamic cellular response to inflammatory stimuli.

## Background

The nuclear factor-κB (NF-κB) transcription factor is ubiquitously expressed in mamallian cells and regulates the expression of many target genes. In the nervous system NF-κB is known to play a key role in the immune and injury responses and in governing normal brain function [1]. During cerebral ischemia NF-κB is a primary regulator of the inflammatory response to ischemic injury, affecting cell death and survival [2]. Microglia, the resident immune cells in the brain, are activated following ischemia and play a controversial role in this decision. Microglia respond to injury in part by releasing both cytoprotective and cytotoxic signaling molecules to surrounding cells, many of which are regulated by NF-κB [3]. As the dynamics of NF-κB activation control gene expression [4-6], characterizing the dynamics of NF-κB activation in microglia is of great interest.

**Figure 1:**
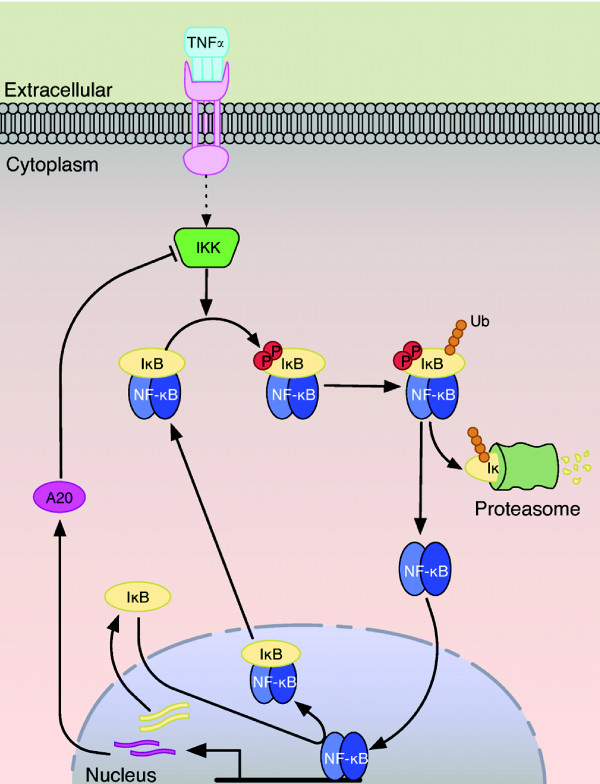
**The canonical NF-κB activation pathway**. Binding of TNFα trimers to TNFR receptors initiates the canonical signaling pathway by activating the upstream kinase IKK. IKK phosphorylates the IκB inhibitor that is bound to NF-κB in the resting state. This targets IκB proteins for the ubiquitin-proteasome system, which leads to IκB destruction by the 26S proteasome and release of NF-κB. Free NF-κB enters the nucleus and activates gene expression of many target genes and induces negative feedback regulation by synthesizing IκB and A20. IκB proteins inhibit NF-κB activity by sequestering NF-κB from the nucleus to form an inner feedback loop, while A20 attenuates stimulus induced IKK kinase activation further upstream in an outer negative feedback loop.

Members of the NF-κB family of transcription factors are found in their inactive state as dimers bound to their IkB inhibitor proteins. Upon stimulation by a diverse set of stimuli, NF-κB is freed from its inhibitor to coordinate gene expression in a highly specific and tightly regulated manner. The IκBα inhibitor and p65(RelA):p50 NF-κB heterodimer are the most extensively studied members of their respective families, and their response to extracellular stimuli illustrates the canonical pathway of NF-κB activation (Figure 1).

In the canonical pathway, binding of extracellular TNFα trimers to TNFR1 receptors at the cell membrane initiates NF-κB activation. The ligand-receptor complex interacts with several adapter proteins, including TNF receptor-associated factor 2 (TRAF2) and receptor-interacting protein-1 (RIP1), which are essential for recruitment and activation of the IκB kinase complex (IKK) [7]. The IKK complex involved in canonical NF-κB activation is composed primarily of the regulatory subunit IKKγ (NEMO) and two catalytic subunits: IKKα/IKK1 and IKKβ/IKK2. Upstream signals activate IKK by phosphorylation of the kinase domain of IKKβ, which in turn phosphorylates IκBα on serines 32 and 36 [8]. Phosphorylated IκBα is recognized by the βTrCP containing Skp1-Culin-Roc1/RBx1/Hrt-1-F-box (SCF) E3 ubiquitin ligase complex (SCF-βTrCP), which facilitates K48-linked polyubiquitination of IκBα and targets it for degradation by the 26S proteasome [9,10].

NF-κB is released following proteasomal degradation of IκBα [11] and translocates to the nucleus, where it activates gene expression. Of the hundreds of genes targeted by NF-κB [12], two in particular are ikba and a20. The expression of these genes is rapidly induced by NF-κB and triggers the synthesis of *de novo *IκBα and A20 proteins. Newly synthesized IκBα sequesters NF-κB from the nucleus to inhibit further transcriptional activity, forming a strong negative feedback regulatory mechanism. The synthesis of A20 proteins creates a second negative feedback loop by regulating the ubiquitination of adapter proteins responsible for activating the IKK complex, thus inhibiting further NF-κB activation [13]. Many characteristics that define TNFα induced NF-κB activation also underlie cellular responses to many other stimuli, necessitating a thorough understanding of this pathway.

Given the dynamic nature of NF-κB signaling and its regulation involving multiple feedback loops, it is necessary to consider the network as a whole when studying this system. The seminal work by Hoffmann and colleagues [4], in which simulation predictions were used in coordination with experimental studies of IκB knockout cells to reveal functional differences among three IκB isoforms, established mathematical modeling as a vital tool for studying NF-κB signaling at a systems level. Subsequently a number of researchers have used modeling to investigate various aspects of NF-κB activity [5,6],[14-18].

Here we develop a mathematical model to describe NF-κB signaling in microglia. Beginning with a recently published model structure shown to be capable of predicting NF-κB signaling in other cell types [14], we attempt to identify model parameters to match experimental data sets of NF-κB and IKK activation obtained from a microglial cell line. The inability of the original model to recapitulate NF-κB activation that is consistent with experimental data -- regardless of model parameter choice -- leads us to expand the model to incorporate previously unmodeled dynamics of the IκBα ubiquitin-proteasome degradation pathway. We also find that IKK activation in microglia is highly nonlinear, which prompts refinement of the upstream signaling module. We use the new model to predict the levels of another network component, total IκBα, and are able to validate this prediction experimentally. The results offer a validated model that can be used as a new tool to study the dynamics of NF-κB activation in microglia. While we find that many key features of canonical NF-κB activation are shared in microglia, the model suggests a potentially more prominent role for the ubiquitin system in regulating the dynamics of NF-κB activation. We use numerical analyses of this model to gain insight into how microglia regulate both IKK and NF-κB activity in response to inflammatory stimuli. Our sensitivity anlayses emphasizes the dynamic nature of how key system responses are regulated, a feature that may not be apparent from similar analyses. The analysis further highlights the robust yet fragile nature of the NF-κB signaling pathway due to the multiple layers of feedback regulation.

## Results

### TNFα stimulates dynamic NF-κB and IKK activation in BV2 microglia

**Figure 2:**
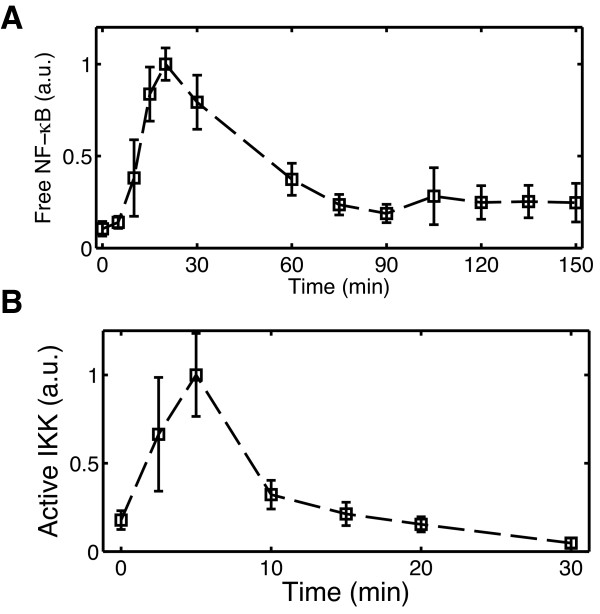
**Dynamics of NF-κB and IKK activation in BV2 microglia treated with TNFα**. ELISA measurements of (A) NF-κB p65 DNA binding activity and (B) IKKβ kinase activity following continuous stimulation by 10 ng/ml TNFα. Data markers at each time point are sample averages from independent experiments performed on separate days. Error bars indicate one standard deviation of the samples.

To characterize the dynamics of canonical NF-κB activation in microglia, cells from the microglial cell line BV2 were cultured and treated with 10 ng/ml TNFα. Whole cell extracts were collected in triplicate over a time course following stimulation in five identical experiments conducted on different days. ELISA measurements of NF-κB p65 DNA binding activity show that NF-κB activation in BV2 microglia is strongly induced by TNFα (Figure 2A). Five minutes following TNFα treatment NF-κB activation remains near basal levels but increases rapidly thereafter, reaching maximal activity near 20 min. Following the initial peak, NF-κB activity declines until approximately 90 min when it returns to a second, smaller amplitude peak. The biphasic NF-κB activity profile in BV2 microglia is consistent with the NF-κB response to sustained TNFα stimulation observed from population level measurements in many other cell types [19].

To better characterize the inflammatory response in microglia we additionally examined the activation of the upstream IκB kinase (IKK) experimentally. The time course of IKK activity was measured for the first 30 min following 10 ng/ml TNFα treatment in three identical experiments. IKK is rapidly activated, reaching peak levels near 5 min. By 10 min IKK activity sharply drops to below half-maximal levels and gradually declines to near basal levels over the next 20 min (Figure 2B). This transient profile resembles IKK activation characteristic of the response in most other cell types to high TNFα doses, in which IKK activity peaks between 5-15 min and drops below 25% of its maximal value by 30 min [20,21]. However, the rapid decline from maximum activity at 5 min to ~33% activity by 10 min is particularly prominent in microglia.

### Intermediate steps in the IKK-induced IκBα degradation pathway reconcile the mathematical model with NF-κB activation in microglia

Next we sought to quantitatively describe microglial NF-κB activation using a mathematical model. While a number of mathematical models for NF-κB have been published in recent years (reviewed in [19]), our preference was to begin with a simple description that still captures the essential components of the network. For this purpose we selected a deterministic, ordinary differential equation (ODE) model structure recently published by Ashall et al [14], which was based primarily on an earlier model by Lipniacki et al [18]. This model includes the core architecture of the canonical signaling pathway and was able to predict many key features of NF-κB activation in different cell types under a variety of conditions.

We first attempted to identify parameters for the existing model structure to fit the experimental NF-κB and IKK activation profiles of microglia. An optimization-based parameter estimation algorithm was run using many randomly selected parameter values from the parameter space as initial guesses. However, no parameter sets were found that matched microglial IKK and NF-κB activity. In particular, the model was unable to qualitatively reproduce the rapid induction and attenuation of IKK activity observed in microglia for any of the parameter sets tested, and NF-κB activation was predicted to occur more rapidly than the 5 min delay observed in Figure 2A. The discrepancies between the model and data prompted us to investigate the time interval immediately following TNFα stimulus.

**Figure 3:**
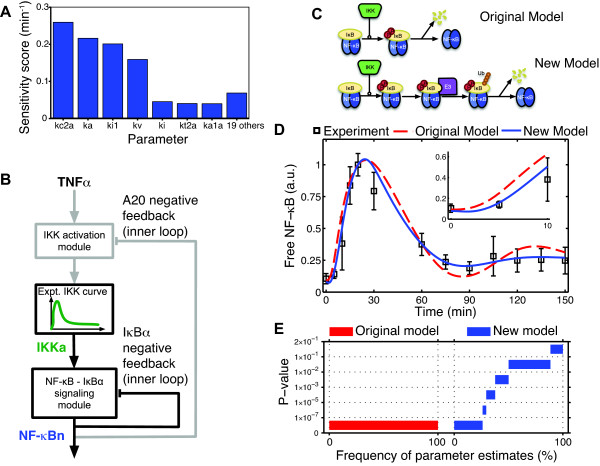
**New model structure required to characterize NF-κB activation in microglia**. (A) NF-κB activity during the first 10 minutes following stimulation was only highly sensitive to seven of the 26 rate parameters. (B) By using an IKK signal derived from experimental measurements as the model input, the outer feedback loop can be removed (indicated by gray lines), isolating the downstream NF-κB activation module with IκBα feedback. Similarly, once the concentration of nuclear NF-κB is known, this signal can be used to drive the upstream IKK activation network independently of the downstream module. (C) Model structure from the original model (top) and the new model (bottom). (D) Simulations with parameters estimated for the existing model (dashed line) and the new model (solid line) using the experimental IKK curve as input. The inset provides a detailed view of the model fits during the initial activation phase. (E) The results of 1980 randomly initialized parameter estimates for each model were checked for statistical consistency with the data using Fisher's Method (see Methods) and binned according to p-value. No estimated parameter sets with the original model achieved a P-value >10^-7 ^(red), while nearly half the estimated parameter sets with the new model (blue) had P > 0.01.

Sensitivity analyses were performed on the model to quantify the relative contributions of each of the system parameters to the concentration of free NF-κB during the first 10 min given the large mismatches between the model and data in this interval. Only seven of the original 26 system parameters have appreciable effects on NF-κB activity during this time based on their time-averaged sensitivity scores (Figure 3A, Additional file 1: Figure S1). Notably, the most significant parameters comprise the rates governing IKK activity, IKK-induced phosphorylation and degradation of bound IκBα, nuclear import of NF-κB and its association with IκBα, and the ratio between the volumes of the cytoplasm and nucleus. No parameters governing transcriptional regulation or other downstream processes have significant effects on NF-κB activation during this early time interval as evidenced by their very small sensitivity scores. Moreover, this ruled out the possibility that feedback from other IκB isoforms (e.g. IκBε) not included in this model could be added to account for the discrepancies in the dynamics. This suggested that the brief delay in the initiation of NF-κB activation observed in microglia was likely due to unmodeled dynamics involved in the IKK-dependent degradation of IκBα or to dynamics in the upstream signaling pathway governing IKK activation, allowing us to restrict our initial attention to only a subset of key upstream parameters.

To more easily explore these possibilities and to facilitate model development, we first considered the downstream network independently of the upstream IKK activation network. IKK interacts with the downstream module only through its enzymatic phosphorylation of IκBα and through feedback inhibition from A20 (Figure 3B). We isolated the downstream network by breaking the outer A20 feedback loop and using the interpolated experimental IKK activation data as the model input in a manner resembling previous work by others [22].

With the IKK profile fixed as the model input, the least squares parameter estimation procedure was repeated with certain parameter values and biological features constrained by the literature (Additional file 1: Table S2). Simulations of the existing downstream model with the estimated parameters predicted free NF-κB levels increasing sooner than what was detected in microglia, as was also the case for the full model (Figure 3D). To test whether this result was limited to a particular set of values or held more generally, many additional estimates were obtained starting from initial values randomly sampled from the parameter space using both a least-squares objective function and an alternative objective function adapted from the parameter estimation method proposed by [23]. Following the methodology in [23], we applied an *a posteriori *statistical test based on Fisher's Method to check whether model simulations at each estimated parameter set were consistent with the experimental data, taking into account measurement errors in the data (see Methods). The results showed that with the original model structure, 100% of the estimated parameter sets had P-values < 10^-7 ^(Figure 3E), leading us to conclude that the original model could not produce dynamics consistent with the data.

Taken together with the sensitivity results showing that very few system parameters significantly affect NF-κB activation during the first 10 min of activation, this strongly suggested there were likely unmodeled dynamics within the IKK-induced IκBα degradation pathway. We next investigated whether the model could be modified in a biologically meaningful way to incorporate missing dynamics and to better fit the data.

The original model structure describes IKK-dependent IκBα degradation in two steps: phosphorylation of IκBα catalyzed by IKK, and degradation of phosphorylated IκBα (Figure 3C). However, this two-step description omits many intermediate steps which occur prior to IκB degradation by the 26S proteasome [7]. We therefore extended the model to include two intermediate reactions following IκBα phosphorylation and preceding IκBα degradation, which we posited might be sufficient to account for the missing dynamics. The reactions roughly correspond to recognition of phosphorylated IκBα by an E3 ligase intermediate, and attachment of a ubiquitin (Ub) chain to the substrate (Figure 3C). It must be noted that each of these reactions potentially encompasses numerous intermediate steps and may not correspond directly to the reactions as they are described here; however, the mechanistic details of this pathway obtained from the literature provide a biological basis for developing this model.

With the new model structure in place, the parameters corresponding to the new stimulus-induced IκBα degradation reactions were estimated using the optimization algorithm while fixing all other parameters downstream of IκBα degradation to their previously estimated values. Remarkably, parameters were found to closely match microglial NF-κB activation, decreasing the data fitting error by nearly 67%, with over a 9-fold improvement during the first 20 min in particular (Additional file 1: Figure S2). Re-estimating the other parameters with the modified model provided even better agreement with the data, further reducing the fitting error from 0.67 to 0.30 (Figure 3D). The consistency between simulations of the new model and the data was assessed using the *a posteriori *statistical test as before. At these parameters the test yielded a P-value of 0.038, implying that the null hypothesis could not be rejected with a high significance level. This result was corroborated by obtaining a large number of parameter estimates and finding that nearly 50% of the estimates with this model structure had P > 0.01 (Figure 3E).

These results provide strong evidence that the addition of dynamics roughly corresponding to the steps involving phosphorylated IκBα recognition and binding by the E3 ligase, polyubiquitination, and proteasomal degradation is sufficient to account for the slightly delayed NF-κB activation observed in microglia.

### Nonlinearities in IKK activation and inactivation produce the rapid transient IKK activity in microglia

We next focused our attention on the upstream signaling pathway governing IKK activation in response to TNFα stimulation. The upstream signaling module was decoupled from the downstream model by using the concentration of free nuclear NF-κB produced by the downstream module as a fixed model input (Figure 3B). This enabled us to consider only the reactions immediately governing IKK activity and its regulation by A20, again greatly simplifying the model development task.

**Figure 4:**
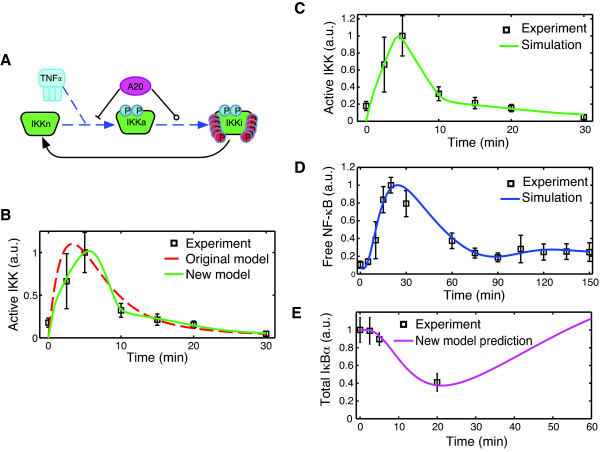
**Development of the upstream model and full model fits**. (A) The upstream model with A20 inhibition at two points was modified to include nonlinear IKK activation and inactivation rates, indicated by dashed lines. (B) Simulations with the newly developed upstream model (solid) provided excellent agreement with the model (P = 0.854, SSE = 0.038), much better than what was possible with the existing model structure (dashed) (P = 0.0002, SSE = 0.661). With the newly developed upstream and downstream modules integrated into the full model, new parameter estimates were able to fit both NF-κB (C) and IKK (D) activity in microglia. (E) Model predictions for the total amount of IκBα (solid line) are compared with experimental measurements of total IκBα protein in BV2-cells following 10 ng/ml TNFα treatment.

The original upstream model in [14], which includes IKK cycling among three states (native, active, and inactive) and feedback from A20, was unable to adequately fit either the rapid activation or deactivation of microglial activation (Figure 4B). Therefore, we examined ways in which the model could be modified consistent with the biology to better correspond with the data.

Activation of the IKK complex at the biomolecular level involves the recruitment and assembly of a signaling complex following TNFα binding to its receptor, as well as numerous post-translational modifications to the complex subunits before IKK is activated by phosphorylation at two residues within its kinase domain [7]. Although other studies have attempted to model the upstream pathway in a greater level of detail [24-26], many of the details are still being resolved and we opted to retain the basic IKK cycling description from [14]. The activation reaction rate was changed from a linear function to a nonlinear Hill equation as a coarse approximation to the many intermediate steps involved in IKK activation.

The quick attenuation of IKK activity following its induction is essential to proper signaling and the resulting biphasic NF-κB activity [20,27]. IKK reportedly undergoes hyperphosphorylation at 9 or 10 residues in the C-terminal, which was found to significantly decrease kinase activity in cells [27]. We posited that potential cooperativity in IKK inactivation due to autophosphorylation may lead to nonlinearites in the inactivation rate equation of the model. Accordingly the linear reaction rate was changed to a nonlinear Hill equation.

Feedback from A20 in the published model was proposed to inhibit the transition of inactivated IKK back to its native state [14]. Because we were unaware of any biological basis for such a mechanism, we adopted two mechanisms of A20 interaction (Figure 4A) that had been identified in the literature and had also been included in prior models. The first is direct inactivation of the IKK complex by A20 protein, a mechanism reported in [28,29] and previously modeled in [18]. We used the identical mathematical description of this interaction from [18] in our model. Secondly A20 is known to inhibit activation indirectly through its ubiquitin-editing activities of upstream signaling components [13]. This mechanism has been included in previous models that have a more detailed description of the upstream signaling pathway [25,26]. We adapted this second interaction to our model by assuming that A20 attenuates the rate of TNF-induced IKK activation in a concentration dependent manner.

Parameter estimation was performed using the newly developed upstream model with fixed nuclear NF-κB as the model input. Parameters were found for which the model produced excellent agreement with microglial IKK activation (Figure 4B), decreasing the fitting error by more than an order of magnitude compared to the best fit achieved with the original upstream model (error 0.04 compared with 0.66). Consistency of the predictions using the new upstream model with the IKK data is more statistically significant (P = 0.85 compared to P = 0.0002) than with original model structure from [14]. Remarkably, we observed that the best fits with the new model were achieved with high Hill coefficients (>3) for IKK inactivation, suggestive of a highly cooperative mechanism in the underlying biological process (Additional file 1: Figure S3).

The newly developed upstream and downstream signaling modules were integrated to form the full model characterizing both IKK and NF-κB activity in response to persistent TNFα stimulus (Additional file 1: Tables S1-S3). Model predictions using the parameter sets estimated from the isolated signaling modules, while giving good agreement during the first 30 min, predicted a higher amplitude second phase of NF-κB activity (Additional file 1: Figure S4), which was inconsistent with the data (P < 10^-6^). Numerical investigation showed this more oscillatory behavior predicted by the integrated model was due to small changes in the later activation profile of IKK predicted by the upstream model, which had been assumed to remain at a constant, low level when developing the isolated downstream signaling module. After increasing the rate of IκBα nuclear import and re-estimating the A20 feedback and IKK recycling rates, the newly developed model was able to provide good agreement with the data, with fitting errors of only 0.34 for NF-κB (P = 0.013) and 0.43 for IKK (P = 0.291) (Figure 4C and 4D).

### Model prediction validated experimentally

Given that the model was developed using a limited set of data from IKK and NF-κB activation, we next sought to test its ability to predict the dynamics of other model species for which no information was used during parameter estimation. The model was first simulated to obtain the levels of total cellular IκBα protein following TNFα stimulus (Figure 4E). The model predicted that the level of protein stays relatively unchanged during the initial delay, but begins a decline by 5 min. At 20 min, the model predicts that IκBα protein levels have been reduced beyond half of their initial amounts.

To test this prediction experimentally, BV2 cells were again treated with 10 ng/ml TNFα, and levels of total cellular IκBα were measured at several time points after treatment using ELISA. The results of the experiments were normalized with respect to the initial quantities and compared with the simulation predictions (Figure 4E). The experimental data were in excellent agreement with the predicted IκBα levels, providing a level of experimental validation to the model.

### Model analysis highlights robustness properties of the network and a dynamic role of feedback regulation in both NF-κB and IKK signaling

The model was next analyzed using sensitivity analysis to gain deeper insight into how the different components of the system interact to regulate the dynamic NF-κB response in microglia. Sensitivity analyses of the NF-κB regulatory network have been performed previously [30-33], and have provided significant contributions to understanding how the system operates. Here we expand upon these studies by considering the dynamic trajectories of the sensitivity coefficients, and examining how the sensitivity of the system response with respect to network parameters changes with time.

**Figure 5:**
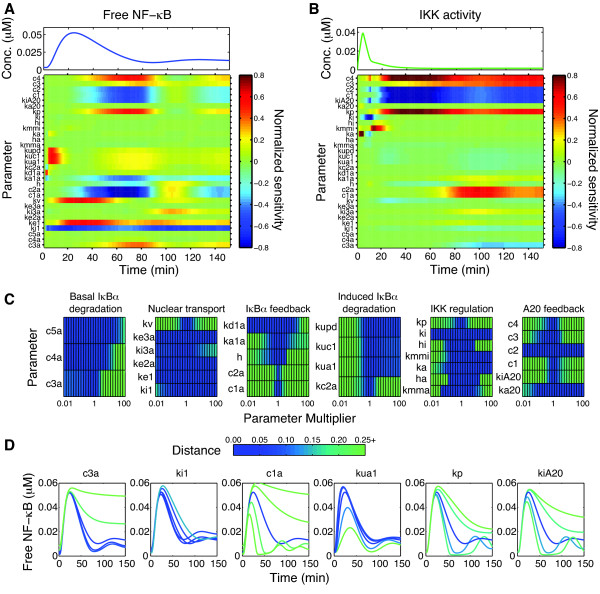
**Model analysis of transient IKK and NF-κB activation in microglia**. Sensitivity analysis of the model shows the dynamic NF-κB response (A) and IKK response (B) are regulated differently by different groups of parameters depending on the time interval of interest. See Additional file 1: Tables S2-S3 for complete descriptions of the parameters. (C) Parameter scans were used to find the Euclidean distance between the nominal and perturbed NF-κB responses as the parameters varied over four orders of magnitude. Dark colors indicate little change, while lighter colors indicate large changes. (D) Simulated trajectories from the parameter scans for selected parameters from each group show distinctions in how the system is affected by up to +/- 10-fold changes from the nominal values. See also Additional file 1: Figures S7 and S8.

The normalized sensitivity coefficients for NF-κB activation were solved and plotted as heat maps to illustrate the dynamic relationship between the signaling components and the system response (Figure 5A).

The sensitivity results clearly show that the NF-κB response is nearly completely insensitive to variations in some rate parameters (rows of light green), but also moderately or highly sensitive to others (dark red and blue) (Figure 5A), consistent with earlier results which found that only a relatively small number of network parameters signifcantly influenced NF-κB activity [30]. A notable feature of our analysis is that, with the exception of the NF-κB nuclear shuttling rates (*ki1 *and *ke1*) for which the sensitivity scores remain high throughout the entire response, NF-κB activity exhibits highly dynamic sensitivity with respect to most other parameters. In other words, there is a strong temporal component to the regulation of NF-κB activity, where variations in different parameters can exhibit great influence over certain phases of activity but have only marginal effects on activation during other time intervals (Figure 5A and Additional file 1: Figure S6). The first 20 min of NF-κB activity is predominantly influenced by the rates for IKK-induced phosphorylation, ubiquitination and degradation and also IKK activation, with little contribution from the feedback parameters. As IκBα is degraded and free NF-κB ascends towards its maximal activity, the nuclear shuttling rates of free NF-κB have the greatest effect.

However, the system shows extreme sensitivity to rates controlling the inner and outer feedback loops. The system is very senstive to the rates for induced IκBα synthesis and its association with NF-κB during a time period coinciding with the decline of the first peak, with synthesis and binding rates negatively affecting NF-κB activation. The rate of conversion of inactivated IKK back to native IKK (*kp*) also is among the most significant parameters in the attenuation of NF-κB activity. While NF-κB activity is at its lowest levels between 60-90 min, the stability of the remaining IκBα transcripts and the induced phosphorylation, ubiquitination and degradation of IκBα exert more influence on free NF-κB levels. The second peak of NF-κB activity is regulated greatly by the nuclear import rate of free IκBα, as evidenced by the high sensitivity of *ki3a *only during this time period. Feedback from IκBα again has highly significant contributions to the dynamics of the second peak, with induced synthesis of IκBα and its affinity to unbound IκBα having very high sensitivities.

The NF-κB response is also highly sensitive to the outer A20 feedback loop in a time-dependent manner. The rates for IKK inactivation by A20 significantly affect the termination of initial NF-κB activity as well as the second phase of activity. This effect is actuated through inhibition of the activation of IKK that has recently been converted from the inactive form and made available for activation; feedback from A20 inhibition of IKK activation has a less substantial role on the dynamics in the model. The outer feedback parameters governing A20 act in opposition to the IKK recycling rate (*kp) *to regulate this response, made clear by the opposite signs of sensitivity values throughout the response.

Although many features of the NF-κB response have been studied previously using sensitivity analysis, little attention has been paid to the dynamic sensitivities of IKK. We therefore assessed parameter sensitivities of IKK activation in the same way as just described for NF-κB (Figure 5B). IKK activity is sensitive to fewer parameters than NF-κB, which is expected due to fewer reactions involved in the upstream module, and its only direct interaction with the downstream signaling pathway occurring through feedback from A20. As with NF-κB, the IKK sensitivities are also highly dynamic, emphasizing the dynamic nature of its regulation during the initial transient and late, low-activity phase. The initial peak only exhibits sensitivity to the activation rate (*ka*) and inactivation rate parameters controlling the magnitude (*ki*) and the dissociation constant (*kmmi*). Twenty minutes after the initial stimulus when IKK is mostly in its inactivated form, the response becomes highly sensitive to the IKK recycling rate and to A20 synthesis, degradation, and negative feedback rates which constitute the outer feedback loop. The late phase IKK response is also relatively sensitive to the rates governing IκBα induced synthesis and transcript stability, and to a lesser extent to its induced degradation of IκBα protein, which indicates that the dynamics of IKK are still highly coupled to the inner feedback loop of IκBα despite the absence of direct crosstalk reactions.

While sensitivity analysis with respect to small variations is informative, the nonlinear nature of the system makes it possible that the results may be different when large magnitude changes to the parameters are considered [30]. Robustness of the system response to large changes in parameter values was therefore assessed by varying each parameter over four orders of magnitude and computing the Euclidean distance between the nominal NF-κB response and the NF-κB response simulated at these perturbed parameters (Figure 5C and 5D). NF-κB activity remains relatively unchanged when many of the parameters for nuclear shuttling and IκBα protein degradation are changed to values which differ substantially from their estimated values, indicating that the system response is relatively robust to changes in these parameters (Figure 5C). Examination of the trajectories at parameter values spanning two orders of magnitude shows that indeed the response remains similar when the protein degradation rates are varied by large amounts, and that altering the nuclear import rate of IκBα changes the amplitude of the second peak but retains an otherwise similar profile (Additional file 1: Figure S7). Consistent with the sensitivity results (Figure 5A) in which NF-κB was insensitive to activation and inactivation rates for IKK, the NF-κB response is robust to changes in these parameter values (Figure 5C). Only extremely large changes in the IKK activation rate parameters significantly alter the response, with much higher activation rates leading to a more oscillatory response (Additional file 1: Figure S8). The parameter scans also show that the system tolerates up to 5-fold changes in the new IκBα induced ubiquitination and degradation parameters while maintaining a similar NF-κB response, but with the timing of the first peak slightly shifted (Figure 5D and Additional file 1: Figure S7). Decreasing the rate further, however, decreased the amplitude of the response significantly. Surprisingly the system is relatively robust to the nuclear import and export rates (*ki1 *and *ke1*), a result which is unexpected given the sensitivity analysis results in which these rates were among the most sensitive. Large changes in these parameters alter the level of damping in the second phase of the response, but the initial peak remains nearly identical (Figure 5D and Additional file 1: Figure S7).

While the system response is robust to large changes in many of the parameter values, the system is much more responsive to changes in the reaction rates involved in both the inner IκBα and outer A20 feedback loops. In particular, the NF-κB activation profile changes significantly when the rates of induced transcription or translation are changed only a small amount, as indicated by the large distance between the nominal and perturbed trajectories at these values (Figure 5C). Changes in these parameters by 3-fold significantly alter how quickly the response is attenuated and change the frequency of the second phase of activity (Figure 5D and Additional file 1: Figure S7). Similarly, the distance remains small for only a relatively narrow range of rates near the nominal values for most A20 feedback parameters, indicating that the system response changes appreciably when these rates deviate substantially from their nominal values (Figure 5C). Large changes in the A20 feedback loop parameters significantly alter both the amplitude and timing of the second peak and how quickly the first peak is attenuated, but leave the early dynamics relatively unchanged (Figure 5D and Additional file 1: Figure S8).

## Discussion

Our quantitative experimental studies show that microglia share many general features of canonical NF-κB activation observed in many other cell types [19]. Namely, microglial NF-κB activity exhibits a biphasic profile with a high amplitude first peak followed by a damped lower-amplitude second phase (Figure 2). NF-κB activation begins following a brief delay of nearly 5 min and reaches a peak near 20-25 min, resembling profiles observed in other studies with immortalized mouse embryo fibroblasts (MEFs) (see, e.g. [4,34]). The second phase of activity appears to be lower amplitude and more heavily damped than that observed in fibroblasts [4,20], although differences in experimental measurement techniques make direct comparison difficult. The observed damping may reflect asynchronous and oscillatory responses at the single cell level [5,35]. IKK activity in microglia also resembles the tightly constrained IKK profile in other cell types that consists of a fast initial peak occurring 5-10 min followed by rapid down-regulation to low levels of activity at later times [20]. One distinction of IKK activation in microglia, however, appears to be the severe attenuation of IKK activity by 10 min following stimulation, only 5 min removed from peak activation levels (Figure 2B).

Despite the general similarities in NF-κB and IKK activation between microglia and other cell types, a recently published mathematical model of the signaling network [14] was unable to recapitulate the nuances of the rapid attenuation of IKK activity simultaneously with the brief delay in the onset of NF-κB activity in microglia. Noting that the largest discrepancies between the data and model simulations occurred within the first 20 min of activation, we used this information together with insight gained from sensitivity analysis to develop a new model that is able to match both IKK and NF-κB activity in this cell type.

The new model was developed in a modular fashion, which was made possible by collecting ELISA-based measurements of IKK in addition to measurements of NF-κB activity and by exploiting the multiple feedback structure of the network. First the IKK data set from microglia was used to develop the downstream signaling module independently of the outer feedback loop, then the upstream signaling pathway was modified to fit IKK activation data, and finally the two modules were integrated to form the full model for which the parameter estimates were refined. The novel downstream signaling pathway includes additional reactions preceding stimulus-induced IκBα degradation, which are sufficient to capture the delayed onset of NF-κB activity observed in microglia (Figure 3D and Figure 4C). The mathematical representation we use to describe the additional dynamics is rather basic, yet captures effects that are likely significant at the biomolecular level. We attribute the intermediate model reactions to key steps in the ubiquitination pathway that implicitly have been lumped together in prior models.

Ubiquitination of IκBα is typically thought to occur almost instantaneously following its phosphorylation by IKK [36]. Consistent with this view, recent *in vitro *kinetic studies revealed in exquisite detail that the SCF-βTrCP E3 ligase sequentially adds ubiquitin (Ub) molecules to phosphorylated substrate to form a polyubiquitin chain able to be recognized by the proteasome in a process lasting only seconds after the first Ub molecule has been added [37]. However, the same study [37] also demonstrated that the addition of the first Ub to the substrate is the rate limiting step and occurs with low efficiency during a single encounter between enzyme and substrate, suggesting that any cellular differences affecting how efficiently the initial Ub is conjugated will contribute appreciably to the dynamics. One such possibility for the differential ubiquitination dynamics is cell-type specific expression of the E3 ligase components, such as the F-box protein, βTrCP, which recognizes phosphorylated IκBα [10]. A smaller pool of βTrCP available to bind IκBα, either as a consequence of reduced expression or increased competition with other substrates such as β-catenin, potentially alters how efficiently the substrate is recognized and hence affects the dynamics. Alternatively differential expression of the two βTrCP isoforms - βTrCP1 and βTrCP2 - may in part account for the altered response in microglia, as studies using genetic knockouts of βTrCP1 found that TNFα induced IκBα degradation was impaired but not prohibited [38]. Others have posited that the unstable βTrCP2 isoform may be stabilized by increased levels of phosphorylated substrate [39], allowing the possibility that microglia express βTrCP2 in excess of βTrCP1 and thereby have altered ubiquitination dynamics.

Besides potentially less efficient recognition of IκBα by βTrCP, another possibility is that the normally rapid polyubiquitination of IκBα occurs less efficiently in microglia due to smaller quantities of Nedd8-ylated Cul-1 in the SCF complex. Conjugation of only a small fraction of Cul-1 with Nedd8 greatly increases the efficiency of ubiquitination of IκBα without affecting the association between βTrCP and phosphorylated IκBα [40] due to facilitated recruitment of Ub-linked E2 to the E3 complex [41]. It follows then that different levels of Nedd8 or the Nedd8-conjugating enzyme, Ubc12, could likely contribute to delayed ubiquitination in microglia. Although we cannot decisively point to a particular mechanism as the source of the additional dynamics needed to match the data in microglia, there are many plausible mechanisms which may warrant further study in the future.

The new model structure indicates a more prominent role of the ubiquitin-proteasome system in regulating NF-κB activation dynamics, which merits consideration of what are its functional implications on how microglia respond to inflammatory stimuli. Analyses of the model show that the ubiquitin-related parameters have large effects on the initial activation of NF-κB and a relatively smaller role in regulating later dynamics (Figure 5A). Parameter scans validate this, as large changes in these parameters change the timing of the first peak by as much as 15 min and alter the amplitude and timing of the later response somewhat (Figure 5C and 5D, Additional file 1: Figure S7). This suggests that altered ubiquitination signaling may be important to regulating the timing of the initial response, but how this affects gene expression and cellular function is not clear at present.

Substantial modifications to the upstream signaling pathway are required to fit the new model to the microglial IKK activation data. The TNFα-induced IKK activation and inactivation reaction kinetics are changed from first order linear mass-action rates to nonlinear Hill equations in the new model. We note that the new model differs from [14] in that it includes mechanisms of A20 feedback that more closely reflect the known biology [13,42], but these mechanisms have also been modeled in previous studies [18,25,26]. The nonlinear reaction rates are essentially black box descriptions of a complex upstream signaling network but allow the model to fit the microglial IKK data remarkably well (Figure 4D). Interestingly, the best agreement with the data is obtained with large Hill coefficients (>3) for the inactivation rate (Additional file 1: Figure S3). This may correspond to cooperativity involved in autophosphorylation at 9 or 10 serines in IKK [27]. Additionally, while autophosphorylation decreases phosphorylation in some cells [27], this effect is not observed in all cells [43], which leaves open the possibility that mechanisms besides autophosphorylation are responsible for the rapid nonlinear deactivation in microglia. Although nonlinearities in the activation and inactivation rates are necessary to match the IKK data well in microglia, they do not appear to have a significant influence on the resulting NF-κB activity, as indicated by our parameter scans (Figure 5 and Additional file 1: Figure S8). Similar findings have been reported elsewhere [20,22,26], and suggest that cells respond robustly to TNFα stimulus by producing an initial peak of NF-κB activity via transient activation of IKK, even in an uncertain environment in which the precise IKK levels may deviate quantitatively but qualitatively remain the same.

In contrast to the parameters governing initial transient IKK activity, our model analyses indicate that the signaling components which regulate later phase IKK activation also exert significant control over NF-κB activation (Figure 5, Additional file 1: Figure S8). Key among these is feedback inhibition by A20, which is known to modulate late phase NF-κB activity through its inhibition of IKK activity [25,42]. Our analysis suggests that direct A20 inactivation of IKK contributes more to later regulation than feedback inhibition of IKK activation, although more detailed models are likely to provide better insight into the complex regulatory role of A20. The analysis also shows that the inner feedback loop of IκBα is significant in later regulation, emphasizing the interconnected nature of the system.

The sensitivity analyses of the new model presented here provide new insights into how this signaling pathway is regulated. In particular, we show by examining the temporal evolution of the sensitivities that there is a strong temporal component to system regulation (Figure 5A and 5B). Previous studies have used sensitivity analysis to identify the key parameters affecting the NF-κB response. These results have typically been reported by ordering the parameters based on the sensitivity scores observed for certain features of the response like the timing and amplitude of NF-κB[30], the L^2^-norm of the dynamic sensitivities [33], or a combination of several dynamic features [32]. While the insights afforded by such analyses are valuable, they can potentially obscure information about the dynamics that are of practical value for model development and parameter estimation. Consider for instance the development of the present model. A reasonable strategy to determine where to modify the model to account for the NF-κB delay might be to start by examining reactions described by the most sensitive parameters as suggested by the literature. However, if these sensitivity rankings are based largely on the effects on later dynamics as opposed to the initial activation, as is the case for all of the feedback parameters, then trying to modify these parameters would be much less effective. Thus, results from our dynamic sensitivity analysis can be of particular importance when trying to identify how to modify a model to correct discrepancies between model simulations and data, as it provides valuable information.

It is important to note that our particular model, which is developed to reproduce population average measurements of IKK and NF-κB activity in microglia, is not unique and other models are capable of producing the same dynamics. It may be desirable in different contexts to extend or otherwise modify this model to explore aspects not considered here. For instance, delayed negative feedback from the IκBε isoform may also contribute substantially to later phase NF-κB signaling dynamics [35,44], but is omitted from the present model. It may be useful to extend the model to include interactions from IκBε in future studies. Using data from bulk population level averages also masks asynchronous NF-κB oscillations at the single cell level [5,14,34,45]. Thus a different approach, such as simulating the deterministic model with random parameter distributions [34] or using stochastic-deterministic hybrid models [6,14,26], may be more appropriate when specifically considering individual cell responses.

The analysis from this model for microglial NF-κB activation clearly portrays the canonical NF-κB response on one hand as very robust: cells are able to parse extracellular signals into transient IKK activation to produce a quick and dynamic rise in NF-κB activity, even in the face of uncertainty in many of the reaction rates in both the upstream and downstream pathways. This finding is consistent with sensitivity analysis of related models, in which the response was found to be largely insensitive to the majority of the rate parameters [30]. On the other hand, this analysis reveals the highly responsive nature of the network, evident from the high sensitivity and low robustness of the NF-κB response to changes in the feedback parameters (Figure 5). We note that although previous analyses have identified the sensitivity of the NF-κB response to many of the same parameters identified here [30,32], none appear to have interpreted the importance of such parameters in the context of feedback control systems. The behavior of the NF-κB regulatory network is not unlike that commonly encountered in feedback systems in the engineering world. Consider, for instance, the operation of an amplifier designed to amplify signals in an electronic system. High gain amplifiers with negative feedback amplify signals robustly even when subjected to relatively large changes in feedforward system parameters. But the response is sensitive to feedback parameters, which both permits the system to be finely tuned by selecting proper feedback components, and makes the system vulnerable to failure if the feedback parameters are altered significantly, perhaps as a result of severe damage. This exemplifies the "robust yet fragile" response that is a general characteristic of complex systems with feedback regulation, whether in biology or engineering [46].

In the NF-κB signaling network, feedback from IκBα-induced transcription allows the system to respond robustly to stimuli to control gene expression, but at the same time makes the system sensitive to changes in feedback parameters. The highly responsive nature of the system makes it particularly susceptible to network perturbations affecting the feedback molecules IκBα and A20, perhaps as might be seen with severe injury such as stroke. However this feature also provides great opportunities for targeted treatment or intervention to modulate the response. Mathematical modeling and analysis may prove indispensible for future exploration of the NF-κB response and drug targeting in microglia, especially when considering crosstalk among multiple pathways that are simultaneously activated by brain injury.

## Conclusions

Mathematical modeling has been used extensively in recent years to provide a detailed view into the activation of NF-κB, helping to make sense of the multiple layers of feedback and to provide a much deeper understanding of how the system functions as a whole. Here we present the development of a mathematical model that quantitatively describes canonical IKK and NF-κB activation in a novel cell type: microglia. The approach we used in model development exploits the multiple feedback structure of the network, and allows the model to be developed in multiple stages by breaking individual feedback loops and developing the modules using the appropriate experimental data. This approach may also prove useful for modeling other biological systems with feedback regulation.

This mathematical model differs significantly from existing NF-κB signaling models in two regards. First, it introduces nonlinearities into the activation and inactivation rates for IKK, which are necessary to reproduce the quantitative IKK profile obtained experimentally and correspond with known biological mechanisms. Secondly, the model includes intermediate dynamics in the induced IκBα degradation pathway. We showed these additional dynamics are essential to characterize NF-κB signaling observed in microglia in a statistically significant manner and are likely due to reactions involved in the ubiquitination and proteasomal degradation of IκBα, suggesting a more prominent role for this system in modulating the NF-κB response.

The mathematical model developed here is the first of its kind for microglia and offers a valuable new tool to study inflammatory signaling in this cell type, permitting rapid numerical simulation and analysis. Our numerical analyses emphasize the highly dynamic nature of regulation of the NF-κB network in response to TNFα stimulus, an aspect which has received relatively little attention in prior analyses. While several key parameters play a significant role in modulating the response throughout the entire duration, many others only regulate the response during specific time intervals, such as during the initial activation phase or the oscillatory later phase. The analysis further provides insight into the robustness properties of the system, indicating high sensitivity to feedback parameters, which we note is analogous to the operation of negative feedback systems in engineering.

## Methods

### Cell culture

BV2 cells, a mouse microglia cell line and kind gift from Dr. K. Andreasson at Stanford University, were cultured in Dulbecco's Modification of Eagle's medium (DMEM, GIBCO, cat# 11995) supplemented with 8% Fetal Bovine Serum (Hyclone, cat#SV30014.03), Penicillin (100 U/ml, GIBCO, cat#15140), and Streptomycin (100 μg/ml, GIBCO, cat#15140). Cells were passaged every four days and were used between passages 10-20.

### Measurement of activated NF-κB p65

BV2 cells were seeded at 4 × 10^5 ^cells per well in six well plates 36 hrs prior to treatment with 10 ng/ml recombinant mouse TNFα (R&D systems, cat# 410-MT). Cells were then harvested for protein at the indicated time points with Phosphosafe Extraction buffer (Novagen, cat#71296) supplemented with 0.01 volume Protease Inhibitor cocktail (Sigma, cat# p8340) and 5 mM DTT before use. Protein concentration was measured using the Coomassie Plus assay (Pierce, cat#23236). 25 μg total protein from each sample was transferred to a pre-chilled Eppendorf tube and brought to 25 μl with complete lysis buffer. These aliquots were stored at -80°C until use for activated NF-κB p65 measurement. Active NF-κB was measured using the Trans AM NFκB p65 Transcription Factor Assay Kit (Active Motif, cat#40096) according to the manufacturer's instructions. 20 μg total protein was used for each sample. Three cultures were assayed for each group. Standards were prepared from recombinant p65 (Active Motif, cat#31102).

### IKK measurements

IKK activity was measured by immunoprecipitation of IKK trimers, followed by a kinase assay/ELISA using a modification of the K-LISA IKKβ Inhibitor Screening Kit (Calbiochem, cat# is CBA044). A total of 400 μg protein from each sample was incubated at 4°C 5 hrs with 5 μg goat anti-IKKγ antibody M18 (Santa Cruz Biotechnology, Cat# is SC8256) with shaking, followed by overnight incubation with shaking with 50 μl 2 × diluted Protein G-Sepharose (Sigma, Cat# is P3296) previously washed in complete lysis buffer. Beads were then centrifuged for 5 min at 13,000 rpm 4°C, the post-immunoprecipitation supernatant removed, and beads were washed in the 1 × kinase assay buffer from the K-LISA kit. Beads were then incubated with shaking in an incubator for 1 h at 30°C in 75 μl 1 × kinase assay buffer containing 150 ng GST-IκBα and 1 × ATP/MgCl_2 _mix from the kit. Beads were then centrifuged at 13,000 rpm for 5 min at 4°C, and 60 μl of supernatant was transferred to a well of the glutathione coated 96-well plate provided with the K-LISA kit. Two-fold serial dilutions of the recombinant IKKβ provided with the kit were run as standards according to the kit instructions, but omitting IKK inhibitor. In addition the post-immunoprecipitation supernatant was concentrated 20 × and run to demonstrate that all IKK activity was depleted from the supernatant. In all cases this sample showed no IKK activity. The plate was incubated 30 min at 30°C to allow the GST-IκBα to bind, and subsequent processing was done according to the vendor's instructions. Final concentrations measured were normalized to the total amount of protein used in a given experiment.

### Total IκBα measurement

Total IκBα measurements from TNFα treated BV2 cells were performed using the PathScan Total IκBα Sandwich ELISA kit from Cell Signaling (#7360). BV2 cells from passage 14-18 were seeded at 4 × 10^5 ^cells/ml on day one and treated with 10 ng/ml TNFα on day three. Cell lysates were prepared and ELISA analysis performed following the manufacturer's instructions. Total protein concentrations were measured using the BCA method; 275 μg total protein was used to measure total IκBα at each time point. The experiments were repeated 3 times.

### Analysis of experimental data

Data from each experiment for NF-κB and IKK was normalized relative to the maximum mean level of activity during that particular experiment to account for variations in optical absorbance readings between experiments. The normalized data were then averaged to produce the ensemble average data set used for data fitting.

### Mathematical modeling and simulation

The model, based on the ordinary differential equation two-feedback model in [14], was developed to incorporate intermediate steps involved in the ubiquitination and proteasomal degradation of IκBα, A20 feedback at multiple points, and nonlinear IKK activation and inactivation rates. The model was integrated numerically using MATLAB 7.7.0 (MathWorks) following the simulation protocol used in [14]. Briefly, the system was initialized with concentrations of total NF-κB and IKK, with all other species set to zero (Additional file 1: Table S1). The model was simulated without stimulus for sufficient time to equilibrate the system. Equilibrium concentrations were then used as the initial conditions for simulations with TNFα stimulus present. Active IKK was assumed to be zero during equilibration and to remain constant at a low level of activity at time points beyond 30 min [20,22] for simulations in which the experimental IKK curve was used as input. The IKKa concentration was computed at each time point during simulation using piecewise cubic Hermite interpolation ('pchip') with the *interp1 *function in Matlab. Similarly, nuclear NF-κB was interpolated in an identical procedure from a simulated curve for development of the upstream module. Further details about the mathematical modeling and tables listing all model species, reactions and parameters can be found in Additional file 1 and Additional file 2. The Matlab source code for the ODE model and simulation script are available upon request.

### Statistical evaluation of model simulations

The agreement between model simulations and experimental data was assessed using an approach based on Fisher's combined probability test [47], which is justified as follows. Each experimental sample is assumed to be the sum of the population mean and measurement noise. The measurement noise is assumed to be iid Gaussian with zero mean. Each data point itself is the bulk average of a large number of cells (>10^5^), and so it is assumed that the sample average from this large collection of cells is normally distributed with mean equal to the population average, but that the standard deviation can vary with time. Individual samples are assumed to be independent across experiment replicates and identically distributed with regard to their respective time points. This is justified since all samples are collected from independent cell populations.

Under these assumptions, a two-sided one sample t-test can be used to compare the population mean from the model simulations corresponding to a specific set of parameters, *θ*, to the sample mean from *n_i _*experimental samples collected at time *t_i_*. The null hypothesis that the two are consistent is rejected at a significance level α if the p-value corresponding to the *i*th t-statistic is *p_i_*< α.

Fisher's method combines the information from the individual test results to test the shared null hypothesis that all the *n_i _*experimental samples come from cell populations whose time evolution of the population average is given by the kinetic model. The test statistic for Fisher's method is computed by combining each independent test as follows:

where log denotes the natural logarithm, and *p_i _*are the p-values from the t-tests at *n *time points. Under the null hypothesis, χ^2^_*F *_follows a chi-square distribution with 2*n *degrees of freedom. The shared null hypothesis is rejected at a significance level α_F _confidence if *p*_F_< α, thus giving a statistical basis upon which a candidate parameter set can be rejected or retained.

### Parameter estimation and sensitivity analysis

Parameter values were estimated by minimizing the a cost function based on the goodness of fit between model and data. Two objective functions were used: one which computed the normalized sum of squares error (SSE),

between the model simulations at parameter set *θ*, *y(t_i_,θ), *and observed data points *y_obs_(t_i_)*, where *i *indexes the *n *time points at which data was collected. A second objective function used the chi-square test statistic computed from Fisher's method (χ^2^_F_), an adaptation of the moment-matching algorithm proposed in [23]. The simulated concentrations of NF-κB and IKK were normalized to their respective concentrations at 20 min and 5 min to allow direct comparison with experimental data. Optimization was performed using the *fmincon *constrained minimization algorithm from the Matlab Optimization Toolbox (MathWorks). Lower and upper bounds for the parameter values were taken from the available literature, as specified in Additional file 1.

The normalized first order sensitivity coefficients of the system,

where *y_i _*is a system output and *θ*_j _is the *j*th rate parameter, were solved using the CVODES forward sensitivity solver from the SUNDIALS 2.4.0 software suite (Lawrence Berkeley National Labs). Sensitivity scores were also assigned based on the time-averaged integral of the normalized sensitivity magnitudes,

## Competing interests

The authors declare that they have no competing interests.

## Authors' contributions

MK, RGG and PWS contributed to the design of experiments, PWS developed the mathematical model and performed the simulations, JFE and XS perfomed the experiments with BV2 cells, PWS, RGG and MK contributed to writing the manuscript. All authors read and approved the final manuscript.

## Supplementary Material

Additional file 1 **Supplementary text and figures**. The pdf contains supplementary text describing development of the mathematical model; Tables S1-S3 which list the model species, reactions, and rate parameters; and Figures S1-S8 that provide more detailed simulation results. Click here for file

Additional file 2 **SBML model of microglial NF-κB activation**. This .xml file is an SBML translation of the mathematical model originally developed in Matlab (MathWorks). Simulations correspond to the response of microglia cells following treatment with 10 ng/ml TNFα. Click here for file
