# Supplementary material for: Quantitative characterization and analysis of the dynamic NF-κB response in microglia
Source: BMC Bioinformatics. 2011 Jul 5;12:276. doi: 10.1186/1471-2105-12-276 (PMC3158563; doi:10.1186/1471-2105-12-276)
Supplement: Additional file 1 — Supplementary text and figures. The pdf contains supplementary text describing development of the mathematical model; Tables S1-S3 which list the model species, reactions, and rate parameters; and Figures S1-S8 that provide more detailed simulation results. [file 1471-2105-12-276-S1.PDF]

Supplementary text and figures

Table of Contents

Mathematical modeling
Supplementary Table S1
Supplementary Table S2
Supplementary Table S3
References
Supplementary Figure S1
Supplementary Figure S2
Supplementary Figure S3
Supplementary Figure S4
Supplementary Figure S5
Supplementary Figure S6
Supplementary Figure S7
Supplementary Figure S8

Mathematical modeling

The mathematical model was developed based primarily on the deterministic 2-feedback model published in [1]. In the deterministic setting, a system of ordinary differential equations (ODEs) describes the dynamics of the concentration of each biochemical species in the model. The species, reactions, and reaction rates included in the model are detailed in the following sections and Supplementary Tables S1-S3. The model includes species in both the cytoplasm and nucleus, with no explicit spatial location considered. Most reactions are modeled with standard mass action kinetics; however, certain reactions required nonlinear reaction rate equations and are indicated where appropriate.

To adapt the model for NF-κB activation in microglia, model development was separated into two stages. First, the signaling pathway downstream of IKK activation that includes the synthesis, degradation, and nuclear shuttling of the IκBα and NF-κB species, was separated from the full model by using the experimentally measured IKK activity profile as the input signal and removing explicit consideration of IKK activation and A20 feedback. The kinetics and parameters of this submodule were modified to provide good agreement with microglial NF-κB activation.

For the second stage in model development, the nuclear NF-κB concentration predicted by the newly developed downstream module was used as the input signal for the upstream IKK-A20 regulatory module consisting of IKK activation/inactivation reactions and A20 feedback reactions. This module was then adapted to adequately match experimentally observed IKK activity and incorporated into the full model. The entire model development process is described

below, followed by tables listing completely all the reaction rates, parameters, and kinetic rate laws of the newly developed model.

Development of the downstream IκBα-NF-κB regulatory module

The IKK activation data points obtained experimentally (Figure 2) were scaled and modified for use as the [IKKa] species in the mathematical model. For simplicity, the basal amount of active IKK was assumed to be zero, and was only assumed to become activated following treatment with TNFα. The maximum level of IKKa was assumed to be ~33% of the total IKK pool [2], which was assumed to be the same as the total concentration of NF-κB. The level of active IKK was assumed to remain constant at the low (but nonzero) concentration observed at 30min for all later time points. The IKKa concentration at all times following TNFα stimulus was interpolated from the rescaled IKK input curve during numerical integration.

Because only a sparse data set (whole cell levels of active NF-κB at 13 time points) was available for fitting, several constraints observed throughout the biological literature were imposed on the parameter estimates to try to obtain more realistic estimates. First, it was assumed that following TNFα stimulation IκBα mRNA transcription is induced at least 10 fold higher than its basal level [1, 3]. Secondly, it was assumed that the amount of basal free IκBα (i.e. unbound from NF-κB) constitutes approximately 15% of the total level of IκBα [4]. Finally, the original model in [1, 4] assumed that phosphorylation of free IκBα was five times less efficient than bound IκBα, as proposed by [5]. More recent work by Mathes et al. [6] provided evidence that both free IκBα and IκBα in complex with NF-κB were similar substrates for IKK, and therefore the phosphorylation rates were constrained to be equal to each other in this work, i.e. *kc1a* = *kc2a*.

With these constraints in place, no parameters for the identical model structure as proposed by [1] were able to satisfactorily fit the active NF-κB profile observed in microglia (Figure 2, Figure S2). A search over many randomly selected initial conditions showed that the model was incapable of correctly fitting the low activity level at 5min while also satisfying the other constraints. The model was modified to include additional reaction steps and species occurring between IκBα phosphorylation by IKK and its degradation, as sensitivity analysis (Figure 3A, Supplementary Figure S1) suggested that reactions involved in these steps had the most significant effects on the dynamics during this early time interval. The newly modified downstream IκBα degradation model included four additional intermediate species: E3pIkBa, E3pIkBaNFkB, uIkBa, and uIkBaNFkB. To simplify matters and reduce the number of free parameters in the system, the reaction rates for free and unbound IκBα species were assumed to be identical. Reverse reactions were not included as they were found to have minor contributions to the dynamics during model development.

In the first set of reactions phosphorylated IκBα was assumed to undergo an intermediate reaction, possibly with the E3 ligase SCF-βTrCP, to form an intermediate species which is unable to be degraded either constitutively or by the 26S proteasome.

$$\frac{d}{dt}[\text{pIkBa}] = -\frac{d}{dt}[\text{E3pIkBa}] \quad = \quad -kua1[\text{pIkBa}]$$

$$\frac{d}{dt}[\text{pIkBaNFkB}] = \frac{d}{dt}[\text{E3pIkBaNFkB}] \quad = \quad -kua1[\text{pIkBaNFkB}]$$

The first intermediate complexes were then assumed to undergo a second set of intermediate reactions that changed the IκBα protein into a form able to be degraded:

$$\begin{aligned}\frac{d}{dt}[\text{E3pIkBa}] = -\frac{d}{dt}[\text{uIkBa}] &= -kc1a[\text{E3pIkBa}]\\ \frac{d}{dt}[\text{E3pIkBaNFkB}] = -\frac{d}{dt}[\text{uIkBaNFkB}] &= -kc1a[\text{E3pIkBaNFkB}]\end{aligned}$$

In the final reactions, the ubiquitinated substrate was degraded, freeing NF-κB from the complex.

$$\begin{aligned}\frac{d}{dt}[\text{uIkBa}] &= -kupd[\text{uIkBa}]\\ \frac{d}{dt}[\text{uIkBaNFkB}] = -\frac{d}{dt}[\text{NFkB}] &= -kupd[\text{uIkBaNFkB}]\end{aligned}$$

Phosphorylated IkBa (pIkBa and pIkBaNFkB) was also assumed to be subject to constitutive degradation independent of polyubiquitination.

$$\begin{aligned}\frac{d}{dt}[\text{pIkBa}] &= -c4a[\text{pIkBa}]\\ \frac{d}{dt}[\text{pIkBaNFkB}] = -\frac{d}{dt}[\text{NFkB}] &= -c5a[\text{pIkBaNFkB}]\end{aligned}$$

Estimating only the parameters involved in the modified IκBα degradation reactions while fixing all other downstream parameters to their values estimated using the original model yielded a significantly better fit with the data (sum of squares error (SSE) of 0.67) compared with the original model (SSE of 2.00) (Figure S2, gray and red curves). The improved fit was primarily a consequence of more closely matching the first 10min of NF-κB activation, as the new model achieved a SSE of 0.16 compared with 1.53 for the original model in this interval. The fit was refined by re-estimating additional downstream parameters with the new model, achieving a SSE of only 0.30 for the whole time course and 0.14 during the first 20 min (Figure 3D and 3E, Figure S2, blue).

Development of the upstream IKK-A20 signaling module

For the second stage in model development, the upstream signaling module was considered independently of the downstream dynamics by using the nuclear NF-κB concentration obtained from simulations of the downstream module described above as the input signal.

Parameter estimation using the model structure from [1] was unable to find parameters that fit the IKK activation characterized experimentally in microglia, especially missing the rapid decline 10 min following activity (Figure 3D and Supplementary Figure S3, gray line), prompting modification to the model structure. During the parameter estimation, it was assumed that a significant fraction of the total IKK pool became active, as the 10ng/ml dose of TNFα is known to be at or near saturation [7]. Therefore, any estimates in which less than 33% of the total IKK pool became active were discarded, based on the constraints proposed in [2].

The published model [1] assumes that the total level of IKK remains constant while IKK cycles between three forms -- "native" IKK (IKKn), active IKK (IKKa), and inactivated IKK (IKKi) -- similar to the mechanism proposed earlier by Karin and Ben-Neriah [8]. When stimulated by TNFα, IKKn is phosphorylated at two serine residues to become activated IKKa, which is then able to enzymatically phosphorylate IκBα. IKKa is assumed to be subject to rapid inactivation to become IKKi, likely by means of auto-phosphorylation at 9-10 residues within the C-terminus [9]. Inactivated IKKi is unable to be activated directly, but does constitutively return to its native state, possibly due to constitutive phosphatase activity. It should be noted that this model greatly simplifies the biology in which IKK exists in many different forms corresponding to various configurations of phosphorylation and/or ubiquitination states, each with potentially different kinase activity. However, as many of the details of IKK activation are still being uncovered, we adopted the same three-state representation for the upstream model.

The model in [1] assumed that A20 feedback inhibited the transition from IKKi to IKKn; however there does not appear to be any biological basis for this interaction. Accordingly, the model was modified to include A20-induced inactivation of the active IKK complex (based on observations in [10] and [11]). This A20 interaction was also included in other related models [7, 12]. Additionally, the model was modified to incorporate a second point of A20 feedback by inhibiting activation. This approach, similar to the modeling approach taken by others [2, 7, 13], is consistent with studies identifying that A20 disrupts the signaling complex upstream of IKK phosphorylation, possibly by ubiquitin-modifications of the RIP1 molecule [14].

Inactivation of IKKa to become IKKi was treated as a monomolecular mass-action reaction in the original model [1] and elsewhere [2, 15]. However, auto-phosphorylation of IKK requires hyperphosphorylation of at least nine C-terminal residues to reduce kinase activity [9]. Therefore the reaction rate function was changed to a Hill equation to account for potential cooperativity involved in the process. The modified reaction rate due to constitutive inactivation is thus given by,

$$ki\frac{[\text{IKKa}]^{hi}}{[\text{IKKa}]^{hi}+kmmi^{hi}},$$

where *hi* is the Hill coefficient.

The first order reaction governing IKK activation, though able to provide adequate agreement with the data for other cell types, was unable to fit the initial microglial IKK activation. The activation of IKK involves receptor-ligand binding at the cell membrane and the subsequent assembly of a multi-subunit signaling complex and numerous post-translational modifications to its components [16]. Rather than developing a more detailed model to describe many of these reactions, such as was done with success elsewhere [2], the activation rate was modified to a nonlinear Hill equation as a function of native IKK concentration [IKKn],

$$ka\frac{[\text{IKKn}]^{ha}}{[\text{IKKn}]^{ha}+kmma^{ha}}.$$

With the nonlinear rates for both the activation and inactivation in place, parameters were found to match the IKK data extremely well (Figure 4B). Further investigation showed that the model was only able to match both the 5min and 10min data points with a Hill coefficient greater than 3

for the inactivation rate *hi* (Supplementary Figure S3 (b)), while any Hill coefficient 1 or greater for the activation rate *ha* gave good fits with the data (Supplementary Figure S3 (a)).

Full model description and newly estimated parameters

The new IKK activation model was integrated with the newly developed downstream signaling model to create the full model. Using the same parameter sets that were estimated above, the full model predicted both NF-κB and IKK very well during the first 30 min, but achieved a worse fit for the later phase NF-κB activity (Figure S4(a), (b)). This was determined to be due to small deviations in the later IKK dynamics from the constant low rate assumed while developing and estimating the downstream parameters. After increasing the IκBα nuclear import rate and re-estimating the later activation, the model was able to fit NF-κB with an error of only 0.48 (0.19 during the first 20 min), and an error of only 0.06 for the IKK levels between 2.5-30 min.

The parameters producing good fits for microglial IKK and NF-κB activity are listed in Supplementary Tables S1-S3. It is important to note that this is only one of a number of possible parameter sets that could fit the data equally well, given the underdetermined nature of the system with many parameters and few experimental observations.

Table S1: Model species and initial conditions. Species not included in the model published in [1] are denoted in red.

Species	Symbol	Initial concentration (μM)
Free IκBα protein (cytoplasm)	IkBa	0
Free IκBα protein (nucleus)	IkBan	0
IκBα:NF-κB complex (cytoplasm)	IkBaNFkB	0.08
IκBα:NF-κB complex (nucleus)	IkBaNFkBn	0
IκBα transcript (cytoplasm)	IkBat	0
A20 protein	A20	0
A20 transcript	A20t	0
Free NF-κB protein (cytoplasm)	NFkB	0
Free NF-κB protein (nucleus)	NFkBn	0
Native IKK complex	IKKn	0.08
Activated IKK complex	IKKa	0
Inactivated IKK complex	IKKi	0
Phosphorylated IκBα protein	pIkBa	0
Phosphorylated IκBα:NF-κB complex	pIkBaNFkB	0
Intermediate IκBα protein	E3pIkBa	0
Intermediate IκBα:NF-κB complex	E3pIkBaNFkB	0
Polyubiquitinated IκBα protein	uIkBa	0
Polyubiquitinated IκBα:NF-κB complex	uIkBaNFkB	0
TNFα stimulus	TNF	0 = no stimulus in equilibrium<br>1 = stimulus present

Table S2: Downstream model reactions and parameters. Reactions or rates developed specifically for this model are indicated in red.

Reaction or constant	Rate	Value	Notes and references
Cytoplasm/nucleus volume ratio	*kv*	5.0	Assumed
IkBa + NFkB ↔ IkBaNFkB	*ka1a* (forward)<br>*kd1a* (reverse)	1.0×10^{0} μM^{-1} s^{-1}<br>5.0×10^{-4} s^{-1}	Increased association rate to maximum suggested in [15] to reduce levels of free basal IκBα
IkBan + NFkBn ↔ IkBaNFkBn	*ka1a* (forward)<br>*kd1a* (reverse)	1.0×10^{0} μM^{-1} s^{-1}<br>5.0×10^{-4} s^{-1}	Assumed same as cytoplasmic rates
NFkB ↔ NFkBn	*ki1* (import)<br>*ke1* (export)	1.58×10^{-2} s^{-1}<br>3.16×10^{-4} s^{-1}	Estimated; constrained 8.0×10^{-4} s^{-1} to 9.0×10^{-2} s^{-1} [1, 15]<br>*ke1* assumed to be *ki1*/50
IkBa ↔ IkBan	*ki3a* (import)<br>*ke3a* (export)	1.32×10^{-3} s^{-1}<br>0.66×10^{-3} s^{-1}	Estimated; constrained 1.7×10^{-4} s^{-1} to 1.5×10^{-3} s^{-1} [1, 2]<br>*ke3a* assumed to be *ki3a*/2 [15]
IkBaNFkBn → IkBaNFkB	*ke2a*	1.0×10^{-2} s^{-1}	[1]; very little effect on dynamics when kept small
nNFkB →NFkBn + IkBat	$c1a * [NFkBn]^{h} / (k^{h} + [NFkBn]^{h})$	$c1a = 2.55 \times 10^{-7}$ μM^{-1} s^{-1}<br>k = 0.065 μM<br>h = 2	Estimated; constrained $1.05\text{-}7.7\times10^{-7}$ [1]<br>*h*, *k* fitted in [1]
tIkBa → tIkBa + IkBa	*c2a*	2.58×10^{-1} s^{-1}	Estimated; constrained 1-5 $\times 10^{-1}$ s^{-1} [12]
tIkBa →∅	*c3a*	2.52×10^{-4} s^{-1}	Estimated; constrained $1.05\text{-}7.7\times10^{-4}$ [1]
IkBa → ∅	*c4a*	5.18×10^{-4} s^{-1}	Estimated; constrained $0.96\text{-}1.92\times10^{-4}$ s^{-1} [1]
NFkBIkBa → NFkB + ∅	*c5a*	2.2×10^{-5} s^{-1}	[1]
IKKa + IkBa → IKKa + pIkBa	*kc1a*	1.73×10^{0} μM^{-1} s^{-1}	Estimated
IKKa + IkBaNFkB → IKKa + pIkBaNFkB	*kc2a*	1.73×10^{0} μM^{-1} s^{-1}	Assumed identical for bound and unbound IκBα [6]
pIkBa → E3pIkBa	*kua1*	3.65×10^{-3} s^{-1}	Estimated
pIkBaNFkB → E3pIkBaNFkB	*kua1*	3.65×10^{-3} s^{-1}	Assumed bound and unbound identical
E3pIkBa → uIkBa	*kuc1*	3.65×10^{-3} s^{-1}	Estimated
E3pIKbaNFkB → uIkBaNFkB	*kuc1*	3.65×10^{-3} s^{-1}	Assumed same as unbound rate
uIkBa → ∅	*kupd*	5.0×10^{-3} s^{-1}	Assumed higher than *kua1* and *kuc1* to prevent accumulation of ubiquitinated IκBα
uIkBaNFkB → NFkB + ∅	*kupd*	5.0×10^{-3} s^{-1}	Assumed same as unbound

Table S3: Upstream model reactions and parameters. Red indicates reactions or rates modified or newly added in this model.

**Reaction**	**Parameter**	**Value**	**Notes and references**
	TNF	TNF=0 (no stimulus)<br>TNF=1 (stimulus present)	
IKKn → IKKa (TNFα induced)	TNF**ka**[IKKn]^{ha} / ([IKKn]^{ha} + *kmma*^{ha}) * *ka20*/(*ka20*+TNF*[A20])	*ka* = 3.37 ×10^{-4} μM^{-1} s^{-1}<br>*kmma* = 7.71 ×10^{-3} μM<br>*ha* = 3.78<br>*ka20* = 8.26 ×10^{-3} μM	Estimated; best fits with *ha*≥1
IKKa → IKKi (constitutive)	*ki**[IKKa]^{hi} / ([IKKa]^{hi} + *kmmi*^{hi})	*ki* = 9.23 ×10^{-5} s^{-1}<br>*kmmi* = 1.27 ×10^{-2} μM<br>*hi* = 9	Estimated; best fits with *hi*>3
IKKi → IKKn	*kp**[IKKi]	*kp* = 2.90 ×10^{-4} s^{-1}	Estimated
IKKa → IKKi (A20 feedback)	TNF**kiA20**[IKKa]	*kiA20* = 3.71 ×10^{-1} s^{-1}	Estimated
NFkBn →NFkBn + A20t	*c1** [NFkBn]^{h} /(k^2 + [NFkBn]^{h})	*c1*=2.21 ×10^{-7} μM^{-1} s^{-1}<br>*k* = 0.065 μM<br>*h* = 2	Assumed same as IκBα transcription
A20t → A20t + A20	*c2*	2.58 ×10^{-1} s^{-1}	Assumed same as IκBα translation
A20t→ ∅	*c3*	2.90 ×10^{-4} s^{-1}	Estimated; constrained 2.9-7.7·10^{-4} s^{-1} [1]
A20 → ∅	*c4*	6.0 ×10^{-3} s^{-1}	Estimated

References

1. Ashall L, Horton CA, Nelson DE, Paszek P, Harper CV, Sillitoe K, Ryan S, Spiller DG, Unitt JF, Broomhead DS, et al: **Pulsatile Stimulation Determines Timing and Specificity of NF-κB-Dependent Transcription.** *Science* 2009, **324:**242-246.
2. Werner SL, Kearns JD, Zadorozhnaya V, Lynch C, O'Dea E, Boldin MP, Ma A, Baltimore D, Hoffmann A: **Encoding NF-κB temporal control in response to TNF: distinct roles for the negative regulators IκBα and A20.** *Genes Dev* 2008, **22:**2093-2101.
3. Kearns JD, Basak S, Werner SL, Huang CS, Hoffmann A: **IκBε provides negative feedback to control NF-κB oscillations, signaling dynamics, and inflammatory gene expression.** *The Journal of Cell Biology* 2006, **173:**659.
4. Rice NR, Ernst MK: **In vivo control of NF-kappa B activation by I kappa B alpha.** *EMBO J* 1993, **12:**4685.
5. Heilker R, Freuler F, Vanek M, Pulfer R, Kobel T, Peter J, Zerwes H, Hofstetter H, Eder J: **The kinetics of association and phosphorylation of IkappaB isoforms by IkappaB kinase 2 correlate with their cellular regulation in human endothelial cells.** *Biochemistry* 1999, **38:**6231-6238.
6. Mathes E, O'Dea EL, Hoffmann A, Ghosh G: **NF-κB dictates the degradation pathway of IκBα.** *EMBO J* 2008, **27:**1357-1367.
7. Tay S, Hughey JJ, Lee TK, Lipniacki T, Quake SR, Covert MW: **Single-cell NF-κB dynamics reveal digital activation and analogue information processing.** *Nature* 2010, **466:**267-271.
8. Karin M, Ben-Neriah Y: **Phosphorylation meets ubiquitination: the control of NF-κB activity.** *Annu Rev Immun* 2000, **18:**621-663.
9. Delhase M, Hayakawa M, Chen Y, Karin M: **Positive and negative regulation of IκB kinase activity through IKKβ subunit phosphorylation.** *Science* 1999, **284:**309-313.
10. Zhang SQ, Kovalenko A, Cantarella G, Wallach D: **Recruitment of the IKK signalosome to the p55 TNF receptor: RIP and A20 bind to NEMO (IKKγ) upon receptor stimulation.** *Immunity* 2000, **12:**301-311.
11. Mauro C, Pacifico F, Lavorgna A, Mellone S, Iannetti A, Acquaviva R, Formisano S, Vito P, Leonardi A: **ABIN-1 binds to NEMO/IKKγ and co-operates with A20 in inhibiting NF-κB.** *J Biol Chem* 2006, **281:**18482-18488.
12. Lipniacki T, Paszek P, Brasier AR, Luxon B, Kimmel M: **Mathematical model of NF-κB regulatory module.** *J Theor Biol* 2004, **228:**195-215.
13. Lipniacki T, Puszynski K, Paszek P, Brasier AR, Kimmel M: **Single TNFα trimers mediating NF-κB activation: stochastic robustness of NF-κB signaling.** *BMC Bioinformatics* 2007, **8:**376.
14. Wertz IE, O'Rourke KM, Zhou H, Eby M, Aravind L, Seshagiri S, Wu P, Wiesmann C, Baker R, Boone DL, others: **De-ubiquitination and ubiquitin**

ligase domains of A20 downregulate NF-κB signalling. *Nature* 2004, **430:**694-699.
15. Hoffman A, Levchenko A, Scott ML, Baltimore D: **The IκB-NF-κB Signaling Module: Temporal Control and Selective Gene Activation.** *Science* 2002, **298:**1241-1245.
16. Hayden MS, Ghosh S: **Shared Principles in NF-κB Signaling.** *Cell* 2008, **132:**344-362.

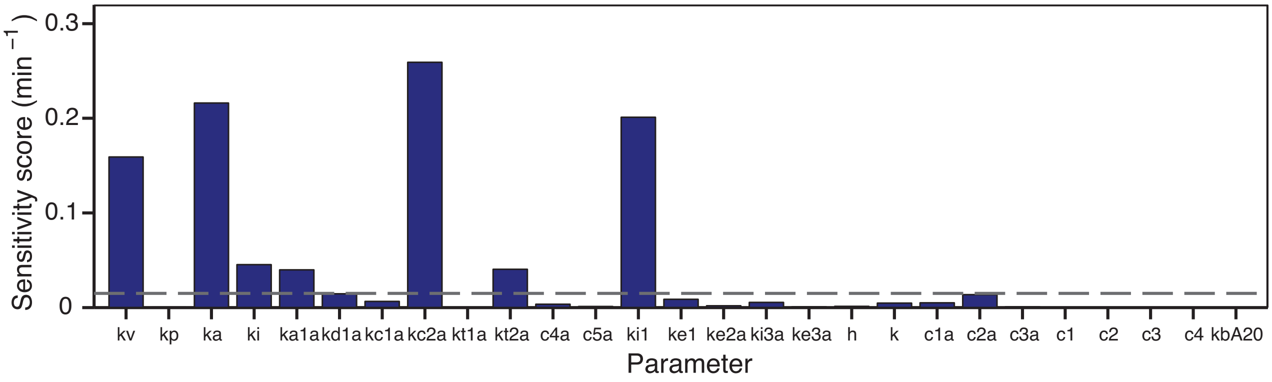

Figure S1: Time-averaged parametric sensitivities during the first 10min of NF-κB activation using the original model from (Ashall *et al.*, 2009). Those parameters whose sensitivity scores were above 0.015 are plotted in Figure 3A.

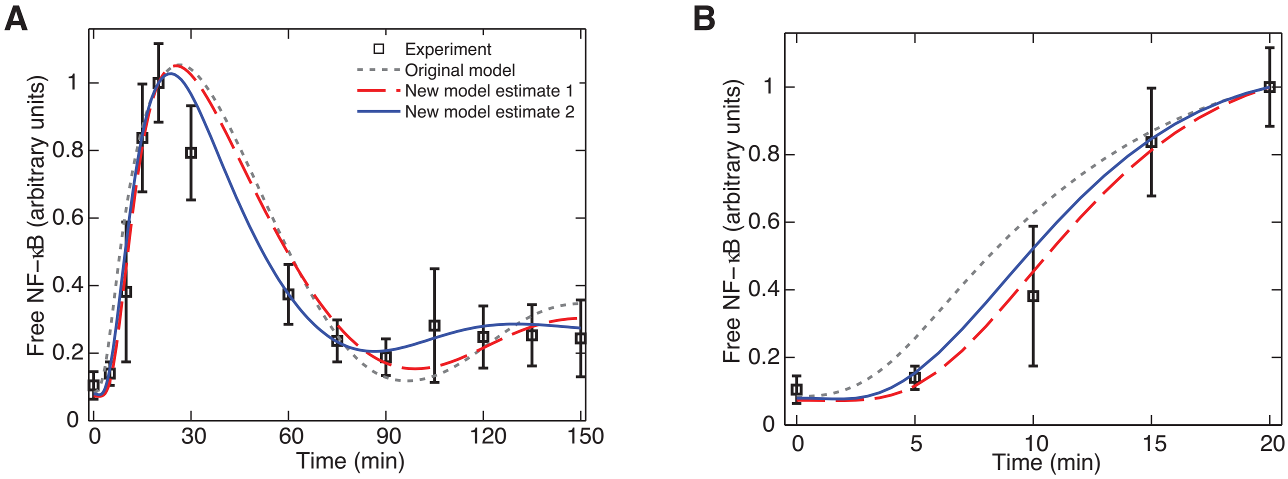

Figure S2: Downstream model development using experimental IKK curve as input. Parameter estimates with the original model (dotted grey line) were unable to produce good fits with the data (black markers) during the first 20 min following TNFα stimulation (error=2.00 during this interval). By introducing additional dynamics to IκBα degradation and keeping all other downstream parameters fixed, the new model is able to match the early dynamics and improve the fit (dashed red line, error=0.16). Re-estimating the downstream parameters with the new model gives a very close match with the data (solid blue line, error=0.13). (B) The same curves as (A) zoomed in to show the fits for the early time points.

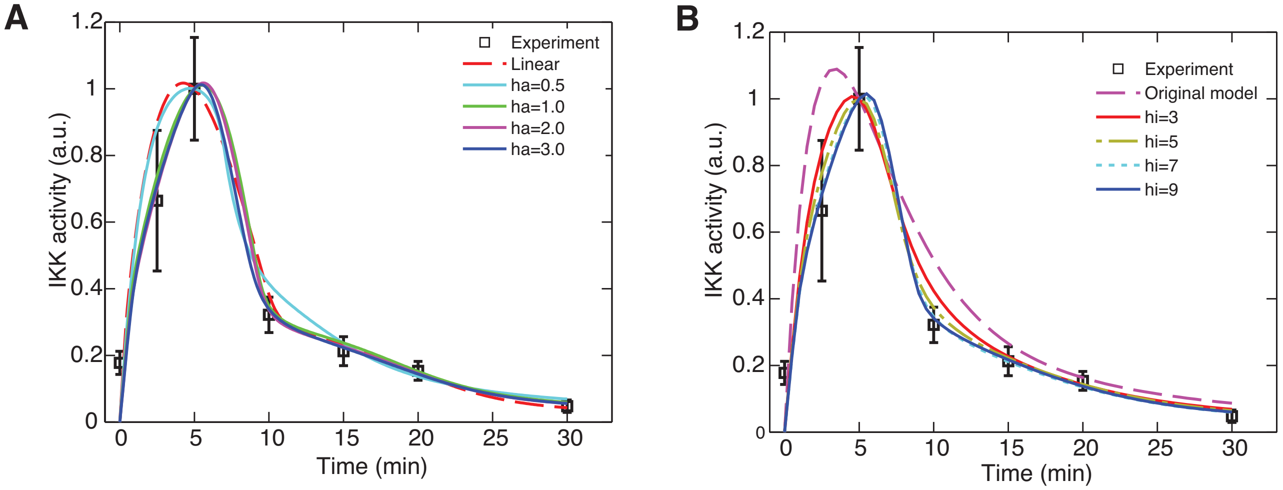

Figure S3: Effect of nonlinearities in upstream IKK activation (A) and inactivation (B) rates.

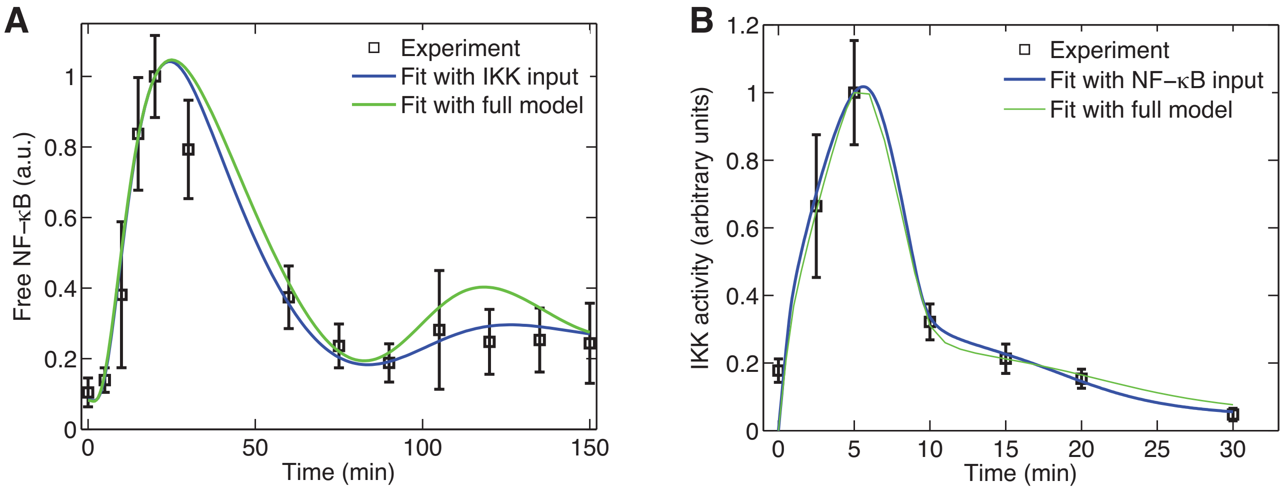

Figure S4: Simulations for NF-κB (A) and IKK activity (B) using the new, full model using parameters estimated when developing the upstream and independent models independently produce slightly worse fits due to small errors in model fitting.

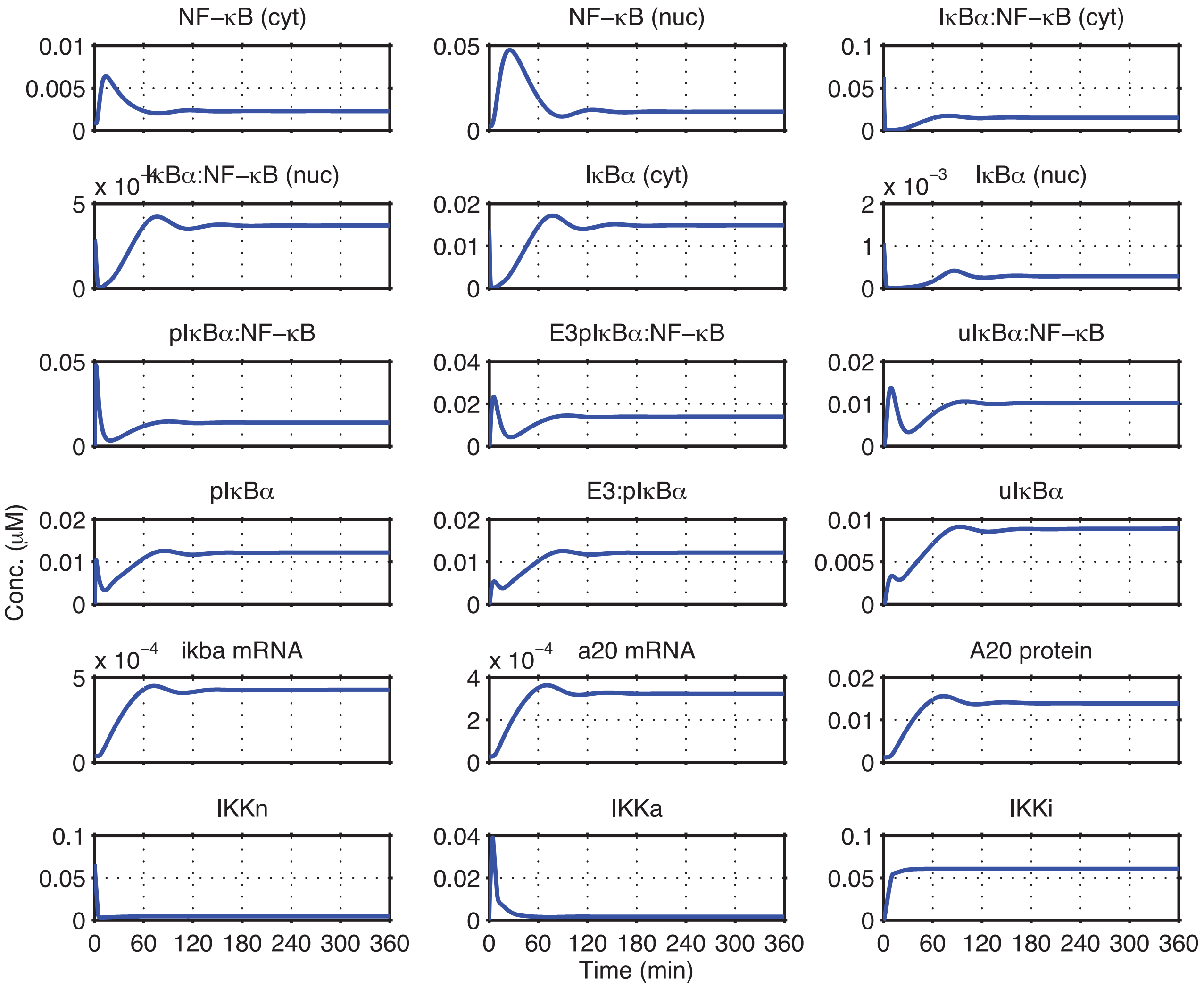

Figure S5: Simulations for all model species with parameter values listed in Supplementary Table S2-S3. Note the vertical axes are scaled differently for each plot.

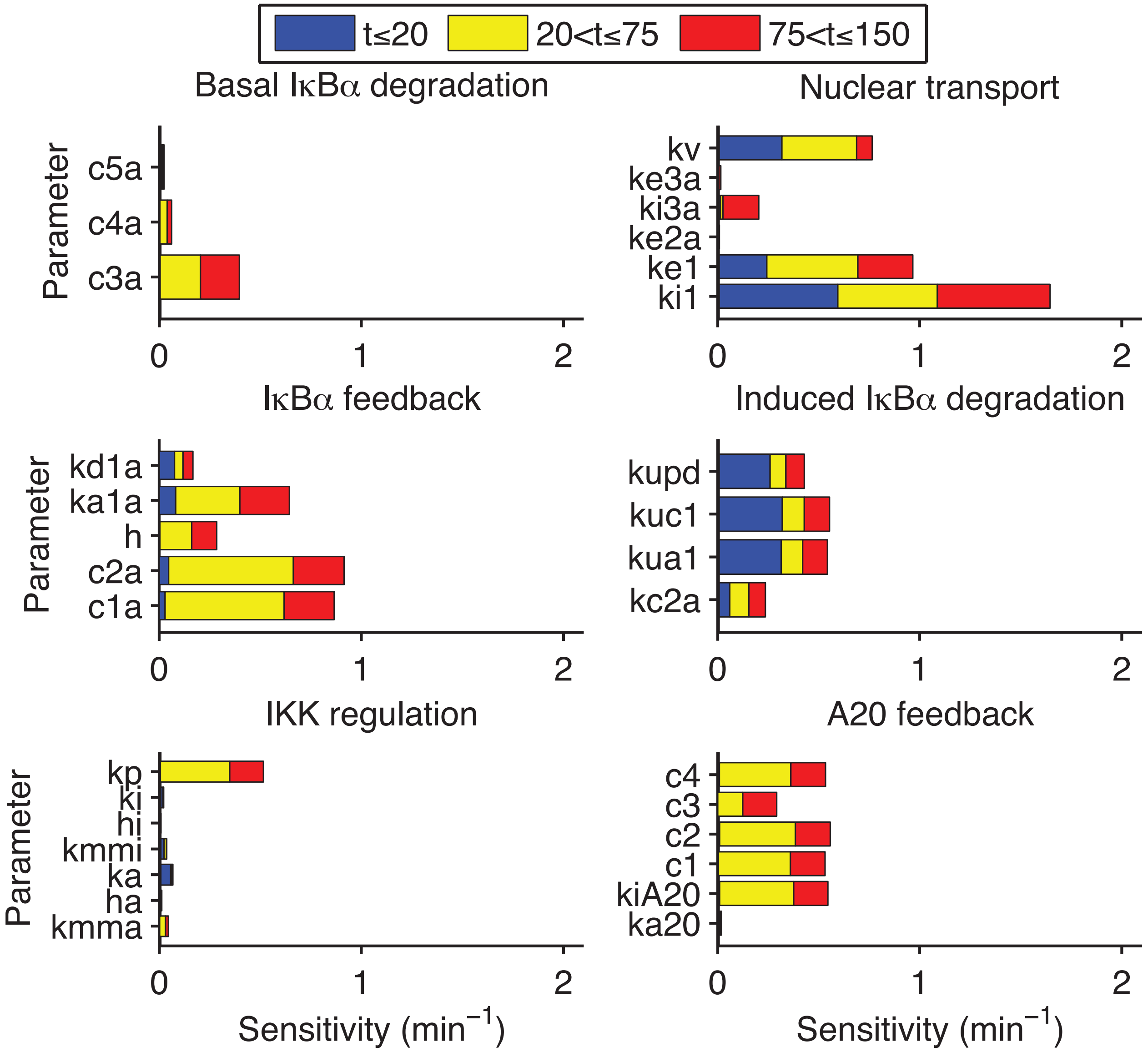

Figure S6: Sensitivity analysis of the microglial NF-κB response. The time-averaged sensitivities of each parameter were computed for three different time periods: 0-20 min (blue), 20-75 min (yellow), and 75-150 min (red). The dynamic nature of regulation is apparent from groups of parameters that have high average scores during some time periods while having little effect on the response during other intervals.

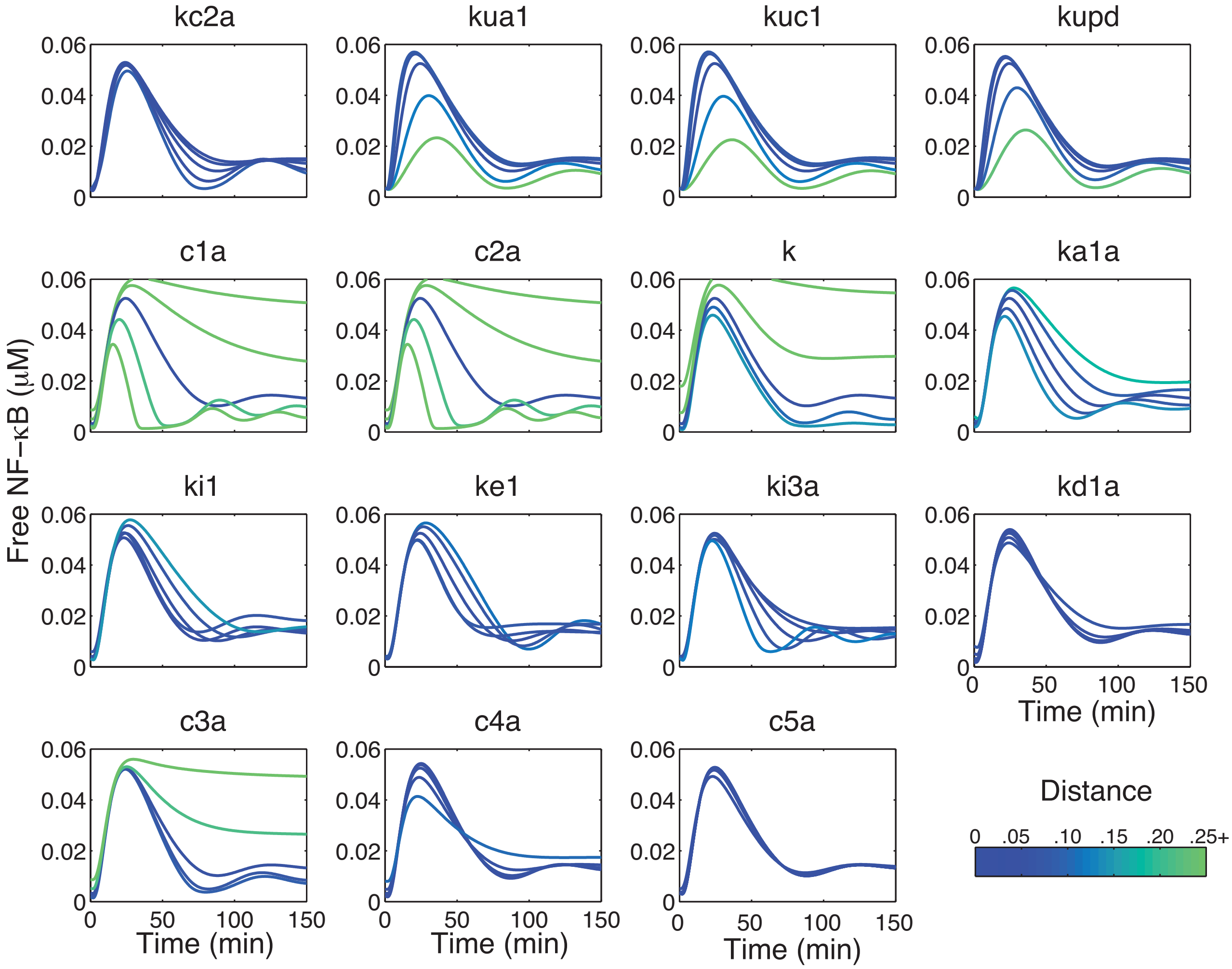

Figure S7: Simulated NF-κB when varying the downstream parameters by 3- and 10-fold their nominal values in either direction. Colors indicate the Euclidean distance between the nominal and perturbed NF-κB trajectories at the perturbed parameter sets.

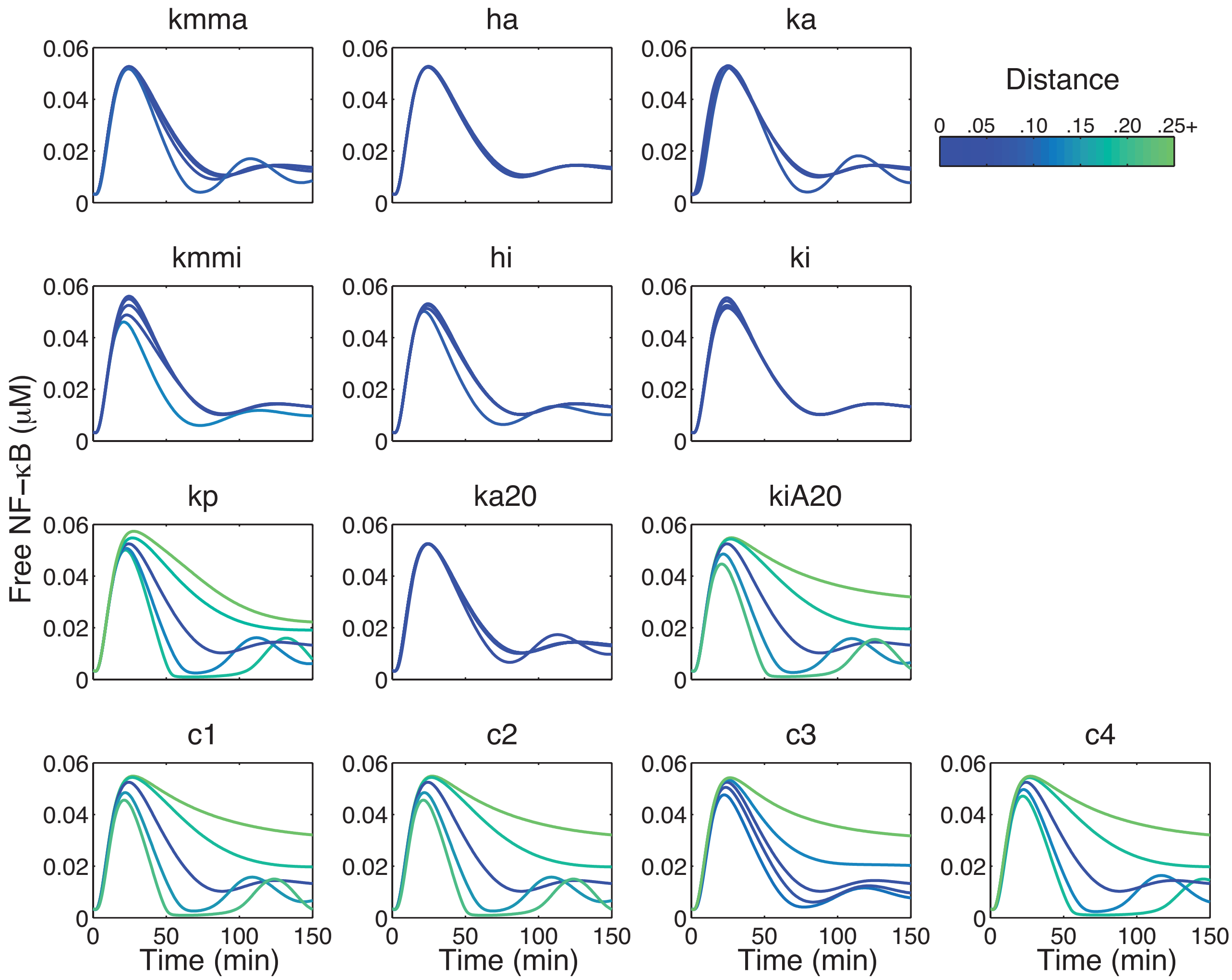

Figure S8: Simulated NF-κB when varying the upstream parameters by 3- and 10-fold their nominal values in either direction. Colors indicate the Euclidean distance between the nominal and perturbed NF-κB trajectories at the perturbed parameter sets.
